# Public health preparedness in times of regional crisis: lessons from Cyprus and the Eastern Mediterranean

**DOI:** 10.1093/eurpub/ckag065

**Published:** 2026-05-19

**Authors:** Alexandros Heraclides, Nicos Middleton, Maria Tsantidou, Andrie G Panayiotou, Ourania Kolokotroni, Souzana Achilleos, Demetris Lamnisos, Eleni L Tolma, Martin McKee

**Affiliations:** Department of Health Sciences, School of Life and Health Sciences, European University Cyprus, Nicosia, Cyprus; Cyprus Epidemiology and Public Health Association (CyEPHA), Nicosia, Cyprus; Cyprus Epidemiology and Public Health Association (CyEPHA), Nicosia, Cyprus; Department of Nursing, School of Health Sciences, Cyprus University of Technology, Limassol, Cyprus; Hellenic Ministry of Health, Department of Oral Health and Dental Care, Athens, Greece; Cyprus Epidemiology and Public Health Association (CyEPHA), Nicosia, Cyprus; Department of Rehabilitation Sciences, School of Health Sciences, Cyprus International Institute for Environmental and Public Health, Cyprus University of Technology, Limassol, Cyprus; Cyprus Epidemiology and Public Health Association (CyEPHA), Nicosia, Cyprus; Department of Nursing, School of Health Sciences, Cyprus University of Technology, Limassol, Cyprus; Cyprus Epidemiology and Public Health Association (CyEPHA), Nicosia, Cyprus; Department of Primary Care and Population Health, University of Nicosia Medical School, Nicosia, Cyprus; Department of Health Sciences, School of Life and Health Sciences, European University Cyprus, Nicosia, Cyprus; Cyprus Epidemiology and Public Health Association (CyEPHA), Nicosia, Cyprus; Cyprus Epidemiology and Public Health Association (CyEPHA), Nicosia, Cyprus; Department of Primary Care and Population Health, University of Nicosia Medical School, Nicosia, Cyprus; Department of Health Services Research and Policy, London School of Hygiene & Tropical Medicine, London, United Kingdom

## Public health preparedness in a volatile region

Public health preparedness is a core element of health system resilience in a world shaped by overlapping crises [1]. Multiple, often interlinked events, including armed conflict, climate change, and population displacement, are being joined by new threats, such as hybrid warfare [2]. The health consequences may be felt far from the areas directly involved. Given these emerging and imminent threats, countries in geopolitically sensitive regions must broaden their approach to preparedness to address direct and indirect consequences for health systems and populations [3].

Cyprus’s recent experience exemplifies these challenges. Following independence in 1960, the United Kingdom retained two sovereign military air bases on the island. In March 2026, soon after the initiation of the US/Israel-Iran armed conflict, one came under a drone attack, most likely launched from nearby Lebanon. It caused limited damage and no casualties [4], but the Republic of Cyprus (holding the Presidency of the Council of the European Union at the time) experienced disruption, including heightened alerts, local school closures, and short-term flight interruptions. Although disruptions were transient [5], there were postponements of international visits and concerns about escalation and indirect economic impacts.

The increasingly interconnected nature of our societies means that contemporary crises can be extremely complex, marked by uncertainty, rapid changes, and indirect effects that extend far beyond the immediate event. Public health impacts stem not only from casualties or displacement but from disrupted logistics, supply shortages, interrupted care, psychosocial strain, and misinformation [1]. Preparedness must reflect this complexity by ensuring systems can absorb shocks and adapt quickly. Public health services must be able to respond proportionately to uncertainty, sustain essential services, and communicate clearly to maintain public trust without fuelling unnecessary alarm.

## The dimensions of public health preparedness

We draw on Cyprus’ recent experience to illustrate the practical measures that health systems can take when regional events generate indirect pressures. These range from ensuring surge capacity and continuity of services to surveillance, psychosocial support, environmental considerations, and intersectoral coordination, highlighting how these elements work together to ensure that health systems remain resilient, responsive, and trusted during fast-moving crises in their wider neighbourhood.

A core component of preparedness is ensuring that health systems can manage sudden surges in demand, with up-to-date emergency protocols and the ability to rapidly expand clinical capacity. These arrangements must be regularly tested in simulation exercises, and clear command-and-control arrangements must be put in place. However, in Cyprus, as in many European countries, maintaining an adequate health workforce remains an ongoing challenge, with nurse shortages especially severe, highlighting the need to incorporate preparedness considerations into broader health workforce planning [6].

Even when health facilities remain intact during a crisis, their ability to deliver care may be affected by disruptions to essential services, as seen during the recent pandemic. Continuity planning must therefore build redundancy into the procurement and distribution of medicines, oxygen, fuel, diagnostics, and other critical supplies. This is especially important for island states, such as Cyprus, that depend on maritime and air links. Contingency plans must include provisions to maintain routine care, including chronic disease management, maternal and child health services, mental health support, vaccinations, and urgent non-crisis care.

Preparedness depends on timely situational awareness. Public health surveillance systems play a crucial role in detecting infectious disease risks, environmental hazards, unusual service pressures, and secondary health effects arising from crisis conditions. In regions characterized by frequent mobility and cross-border interdependence, this function is strengthened by information sharing and coordinated risk assessment. However, early warning systems now need to be conceived more broadly than in the past. Effective preparedness requires the capacity to integrate epidemiological intelligence with environmental monitoring, service-use data, and other indicators, now including a wealth of data from open-source intelligence (OSINT), social media monitoring, and much else [7]. The aim is to detect emerging pressures early enough to support proportionate and evidence-based action.

Preparedness also demands systems that cross borders as easily as the threats they must anticipate, with effective cooperation across jurisdictions [8]. This is now much easier within the European Union but much more difficult on its external borders or, in Cyprus’s case, on an island that has been divided since 1974.

Crises can have both acute and cumulative mental health effects. Prolonged uncertainty, repeated alerts, disrupted routines, and distressing information increase psychosocial needs, even for those not directly exposed to violence. Preparedness must therefore include psychosocial services for vulnerable groups, frontline workers, displaced people, and communities facing ongoing uncertainty. During recent events in Cyprus, authorities offered reassurance regarding security and de-escalation, aided by visible European military support, though some perceived the increased military presence as raising risk, highlighting the complexity of risk perception.

Even minor infrastructure disruptions can affect public health. Interruptions to transportation, communications, utilities, or logistics can hinder access to healthcare and emergency response, while environmental incidents such as fires or chemical releases require rapid coordination. Preparedness must incorporate environmental health expertise, infrastructure contingency planning, and strong links between public health bodies, civil protection, municipalities, emergency services, and transport and communication authorities.

A whole-of-government approach is essential, as crises often originate outside the health sector but quickly create health consequences. Effective intersectoral preparedness requires clear roles, shared protocols, and regular exercises. Recent events in Cyprus, from wildfires to the aforementioned regional conflict, have strengthened cross-sector collaboration and expanded public alert systems such as targeted SMS notifications and the SafeCY app [1].

## Risk communication: maintaining public trust without amplifying fear

Risk communication is a core element of preparedness, shaping behaviour, trust, and institutional legitimacy. Messages that are too vague invite speculation, while overly dramatic ones heighten unnecessary anxiety. Effective communication must be timely, transparent, proportionate, and actionable, acknowledging uncertainty, clarifying what is known, and offering practical guidance without inflaming concerns. In high-volatility information environments, where misinformation spreads rapidly, communication should also stabilize public sentiment and reinforce that preparedness is part of routine governance, not a signal of an imminent crisis.

Developments in Cyprus illustrate two lessons. First, public discussion on shelter capacity highlighted the value of transparency and open communication. Initial governmental statements suggested that existing shelters were broadly sufficient, but scrutiny raised questions about readiness, raising anxiety among the public. A more detailed assessment by the government that acknowledged gaps and outlined plans to expand and upgrade shelter infrastructure reinforced trust in national and local institutions. Second, widespread misinformation, especially on social media, created an exaggerated sense of danger, despite the limited nature of events. As seen during the recent pandemic, unchecked misinformation can distort perceptions. Recognising these risks, authorities acted to provide clear, accessible information through official channels. This helped the public distinguish reliable information from speculation and supported a more resilient information environment. Currently, Cyprus is developing a national strategy for emergency risk communication and community engagement. Meeting these challenges will require strengthened national systems and close coordination with European institutions.

## Regional and European cooperation as pillars of preparedness

No country prepares alone. Regional and European cooperation is essential for public health preparedness, especially in interconnected and crisis-prone regions. Mechanisms such as the EU Civil Protection Mechanism support coordinated prevention, preparedness, and response among participating states [9].

European-level initiatives, including health emergency preparedness structures and cross-border response networks, strengthen national systems through shared planning, stockpiles, logistics, situational awareness, and technical cooperation. These mechanisms are particularly valuable when crises cross borders or place simultaneous pressure on multiple sectors.

For Eastern Mediterranean countries, preparedness is both national and regional. Building resilience requires strong domestic capacities alongside active participation in European and regional frameworks. During recent events in Cyprus, solidarity was expressed mainly through defence measures, with several Member States framing the protection of Cyprus as part of defending Europe and committing military assets. National authorities and the public welcomed this. Building on this tradition of solidarity, similarly rapid and coordinated support could play an important role in reinforcing public health infrastructure and risk-management efforts should Cyprus, or any European country, face a major public health challenge in the future.

## Conclusion: emerging lessons from recent crises—strengthening preparedness

Public health systems must be ready for many types of disruption. Pandemics have shown the need for strong surveillance, coordination, and continuity of essential services. Climate-related emergencies, such as wildfires, highlight the importance of evacuation planning, environmental health capacity, intersectoral coordination, and psychosocial support. Periods of regional tension and armed conflicts further stress the need to manage uncertainty, protect supply chains, and communicate risks clearly.

Preparedness should be routine, not a manifestation of impending instability. The goal is resilient systems that respond swiftly and proportionately to diverse shocks while maintaining essential services, trust, and public confidence.

Recent events in the Eastern Mediterranean, including Cyprus, show how quickly external developments can heighten preparedness needs even without a domestic crisis. The appropriate stance is neither complacency nor alarm but careful planning, institutional learning, and coordinated action. Key lessons recur: preparedness works best when embedded in governance; resilience relies on systems, not individual institutions; indirect impacts can matter as much as direct ones; and public trust is both essential and an outcome of effective preparedness.

Framing preparedness as part of responsible governance reassures partners, residents, and visitors about stability and resilience in a country. A constructive approach focuses on readiness, coordination, and clear communication, strengthening resilience without amplifying fear and protecting both public health and public confidence during regional uncertainty.

Conflict of interest: The authors declare that they have no conflicts of interest.

## Data Availability

No new data were generated or analysed in support of this research.

## References

[1] Cleaver T. SafeCY civil defence shelter finder app fully functional. *Cyprus Mail*. https://cyprus-mail.com/2025/06/14/safecy-civil-defence-shelter-finder-app-goes-live (14 June 2025, date last accessed).

[2] Marchandise C , McKee M. Europe in a hybrid war: health security as strategic defence. Eur J Public Health 2025;35:1074–5. 41240376 10.1093/eurpub/ckaf210 PMC12707500

[3] Correia T , Albreht T, Squires N et al Invisible frontlines: the overlooked health consequences of the war in Iran. Eur J Public Health 2026;36:ckag043. 10.1093/eurpub/ckag043 PMC13017130 41831207

[4] Kambas M , Young S. Iranian-made drone hits British air base in Cyprus. Reuters. https://www.reuters.com/world/europe/british-air-base-cyprus-hit-by-suspected-drone-strike-sky-news-reports-2026-03-02/ (2 March 2026, date last accessed).

[5] Reuters. EU meetings to resume normally in Cyprus from April, Cyprus energy minister says. Reuters. https://www.reuters.com/world/eu-meetings-resume-normally-cyprus-april-cyprus-energy-minister-says-2026-03-16/ (16 March 2026, date last accessed).

[6] Theodorou M , Charalambous C, Williams GA. Cyprus: Health System Review. Copenhagen: World Health Organization, Regional Office for Europe, 2024, 1–146. 40386844

[7] Honeyman D , Gurdasani D, Notaras A et al Global epidemiology of outbreaks of unknown cause identified by open-source intelligence, 2020–2022. Emerg Infect Dis 2025;31:298–308. 39983687 10.3201/eid3102.240533 PMC11845160

[8] McKee M , Atun R. Beyond borders: public-health surveillance. Lancet 2006;367:1224–6. 16631865 10.1016/S0140-6736(06)68520-6

[9] European Commission. EU civil protection mechanism. European Commission, 2026. https://civil-protection-humanitarian-aid.ec.europa.eu/what/civil-protection/eu-civil-protection-mechanism_en (15 March 2026, date last accessed).

